# Twinning across the Developing World

**DOI:** 10.1371/journal.pone.0025239

**Published:** 2011-09-28

**Authors:** Jeroen Smits, Christiaan Monden

**Affiliations:** 1 Nijmegen Center for Economics, Institute for Management Research, Radboud University Nijmegen, Nijmegen, The Netherlands; 2 Department of Sociology, University of Oxford, Oxford, United Kingdom; Africa Centre for Health and Population Studies - University of KwaZulu-Natal, South Africa

## Abstract

**Background:**

Until now, little was known about the variation in incidence of twin births across developing countries, because national representative data was lacking. This study provides the first comprehensive overview of national twinning rates across the developing world on the basis of reliable survey data.

**Methods:**

Data on incidence of twinning was extracted from birth histories of women aged 15–49 interviewed in 150 Demographic and Health Surveys, held between 1987 and 2010 in 75 low and middle income countries. During the interview, information on all live births experienced by the women was recorded, including whether it was a singleton or multiple birth. Information was available for 2.47 million births experienced by 1.38 million women in a period of ten years before the interview. Twinning incidence was measured as the number of twin births per thousand births. Data for China were computed on the basis of published figures from the 1990 census. Both natural and age-standardized twinning rates are presented.

**Results/Conclusions:**

The very low natural twinning rates of 6–9 per thousand births previously observed in some East Asian countries turn out to be the dominant pattern in the whole South and South-East Asian region. Very high twinning rates of above 18 per thousand are not restricted to Nigeria (until now seen as the world's twinning champion) but found in most Central-African countries. Twinning rates in Latin America turn out to be as low as those in Asia. Changes over time are small and not in a specific direction.

**Significance:**

We provide the most complete and comparable overview of twinning rates across the developing world currently possible.

## Introduction

Scientists and non-scientists alike are fascinated by twins. They speak to us about the uniqueness of a human individual and the natural bond between siblings. In some societies twins are revered and in others they are looked upon with suspicion. At the same time, twin studies are fundamental to the scientific understanding of the role of nature and nurture [1], [2]. Yet surprisingly, up to now, we have had a very incomplete picture of the number of twins around the world. Only for highly developed countries with good birth registrations, reliable national information on the incidence of twinning and the changes therein over time is available. Information for less developed regions is weak or lacking all together.

Since the early 1970s, several overview studies have been published in which figures from a large number of smaller studies were brought together [1], [3]–[6]. The overall conclusion drawn from these figures was that natural twinning rates were low in East Asia and Oceania (less than 8 twin births per 1000 births), intermediate in Europe, USA and India (9–16 per 1000 births) and high in some African countries (17 and more per 1000 births). However, for the less developed parts of our world, this conclusion is based on low quality and unrepresentative data for only a handful of countries. Most data were derived from local birth registers, which often are of dubious quality, or from hospital registrations, which are notorious for their selectivity problems. In hospital data, twin births may be either overrepresented, because twin pregnancies are more often associated with complications, or underrepresented, when complicated cases are referred to special hospitals or only first pregnancies are accepted [3], [5]. Comparable and representative national information on the incidence of twin births in developing countries has been lacking up to now.

The current study fills this gap in our knowledge by providing the most comprehensive overview of twinning in the developing world currently possible. This overview is based on a unique data source that has not yet been used for comparative twin research: The large representative household surveys collected since the mid-1980s as part of the Demographic and Health Survey (DHS) programme. Using these data, we compute national twinning rates for 75 low and middle income countries. A comparable twinning rate for China is additionally computed on the basis of published data [7]. For 44 countries, data for more points in time is available, so that changes over time can be studied.

### Sources of variation

Differences in twinning rates among countries and over time are mostly due to variation in dizygotic twinning. Monozygotic twinning is thought to occur at a relatively constant rate of 3.5–4 per 1000 births across human populations [1], [8]–[10]. The most important factor associated with (dizygotic) twinning is maternal age. The number of twin pregnancies increases substantially with maternal age, until age 38, and then decreases again [1], [3], [11], [12]. Other factors associated with twinning are parity (the number of pregnancies experienced by the mother before the twin pregnancy) [1], [12], [13], maternal height [13]–[15], smoking [16], [17], oral contraceptive use [12], [18] and race/ethnicity [1], [6], [19], [20]. There is also a substantial hereditary component, which runs through the female line [21], [22]. A major ‘new’ factor influencing twinning rates across the globe is the increasing use of assisted reproductive technology (ART), like in vitro fertilization (IVF), intra-uterine insemination (IUI) and ovulation induction (OI), which are associated with a higher number of twin births [11], [23]–[25].

Twinning rates in high income countries are known to vary considerably over time [3], [24]–[29]. In the USA, Australia and many European countries (e.g. Austria, Czech Republic, Denmark, Germany, Italy, Norway, Sweden, Switzerland) they decreased from around 12 or higher in the 1920s to under 10 in the 1970s and then increased again to values of 13–16 around the year 2000. The initial decline in these countries was partly caused by a decrease in the age at which women have their children [25]–[27]. In the same way, the recent increase might have partly been caused by the rise in age at childbirth since the 1970s. However, both in Western and in East-Asian countries this increase was also to a considerable part the result of the rise of ART [25], [26], [28]–[30].

## Methods

Data are derived from the Demographic and Health Surveys (DHS), large representative household surveys held since the mid 1980s in many developing countries. The DHS programme is sponsored by USAID and executed by MEASUREDHS, in collaboration with national statistical agencies (www.measuredhs.com). In the DHS surveys all usual resident women aged 15–49 obtain an extensive oral interview in which a complete birth history is collected. Information on all pregnancies that resulted in a live birth was recorded, including whether it was a singleton or multiple birth. We use the birth history data collected in 150 DHS surveys held between 1987 and 2010 in 75 low and middle income countries. To limit the time frame of our figures and prevent selection problems, we restricted our sample to births that occurred in the 10 years prior to the interview. Information was obtained on 2,473,209 births experienced by 1,379,694 women. Of these births, 30,895 were twin births.

As China was not represented in the DHS, we computed a comparable figure for this country on the basis of data published by Gan et al. [7] on women interviewed in the 1990 Census of China who gave birth in 1989. The number of births and twin births in China were computed on the basis of Table 1 of Gan et al. (p. 634). Among the reported 23,477,961 Chinese births, there were 186,273 twin births.

National twinning rates were computed by dividing the number of twin births by the total number of births and multiplying the outcome by 1000. Given the substantial positive association between twinning and mother's age at birth [1], [12], besides these ‘natural’ twinning rates also rates standardized for mother's age at birth are presented. To compute these standardized rates, the age distribution of the countries was transposed to a reference population with an age distribution equal to the average age distribution of the 76 countries included in our study.

## Results

**Figure 1:**
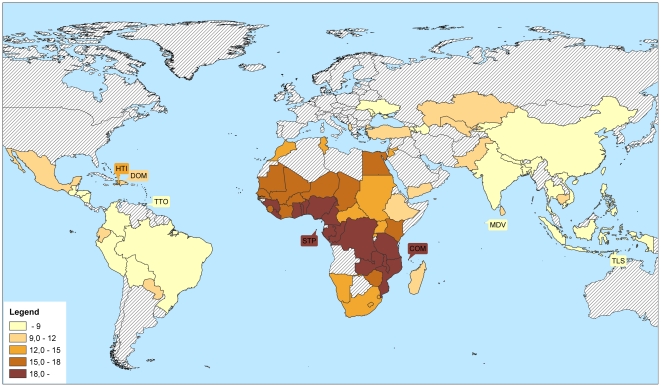
Twins per 1,000 births in 76 low and middle income countries.

The average of the national twinning rates in the 76 countries was 13.1 per 1000 (each country weighted equally) or one twin birth in 76.3 births. This figure is close to the average rate of spontaneous twinning mentioned in the literature of one twin birth in 80 births [5], [10]. National twinning rates for the 76 countries are shown in Figure 1 and in Table S1. The figures are total twinning rates, with monozygotic (identical) and dizygotic (fraternal) twins combined. Given the relative stability of monozygotic twinning rates across human populations [1], [8]–[10], the variation observed among the countries is almost completely due to variation in dizygotic twinning.

Figure 1 shows that there is huge variation in twinning rates across the different regions of the developing world. The very low rates observed for the pre-ART period in Japan, Hong Kong, Singapore and Taiwan [1], [28] turn out to be the dominant pattern in the whole South and South-East Asian region. China, Vietnam, Thailand, Philippines, Indonesia, India, Bangladesh, Nepal and Kyrgyzstan all have twinning rates below 9 per 1000. Twinning rates are somewhat higher in Pakistan, Sri Lanka, Cambodia, Kazakhstan and Uzbekistan, but also in these countries they remain below 10 per 1000 (see also Table S1).

In Africa, Nigeria is dethroned as the twinning champion of the world. The high twinning rates that were known to exist in this country turn out to be the dominant pattern in the whole Central African region. A zone with high twinning rates of above 18 per 1000 runs from Guinea in the West along the Atlantic coast to Congo DR and then crosses the continent to Tanzania, Mozambique and the Comoros. South of this zone, in Namibia, Lesotho, South Africa, and Madagascar, twinning rates are clearly lower with values of 11–15. To the north, we see a gradual decrease, with somewhat lower levels in the Sahel countries and intermediate levels of under 15 in Morocco and Tunisia. A steeper decrease is observed in the north-eastern direction, but there Egypt with a twinning rate of 17.7 is an exception. The highest national twinning rate found in our data is observed in Benin. With 27.9 per 1000, the rate in this country is substantially higher than in any other high-twinning country.

Little was known about the variation in twinning rates among Latin American countries. Our data reveal that this continent to a large extent resembles Asia, with low twinning rates of under 9 per 1000 in most of the countries. Only the Caribbean island Haiti has a substantially higher twinning rate of 14.1 per 1000. This is probably due to the high percentage of persons from West-African descent living there. Bolivia (6.7) is, after Vietnam (6.2), the country with the lowest twinning rate in our data.

There are three middle income Eastern European countries included in our database, Ukraine, Moldova and Albania. Ukraine and Moldova have a low twinning incidence of 8.9 and 8.2 per 1000 births respectively. Twinning incidence in Albania is with 12 per 1000 at an intermediate level. In the Middle East, Turkey has a relatively low twinning rate of 9.9 and Jordan an intermediate rate of 14.4.

Table S1 also presents twinning rates that are standardized for mother's age at birth, the behavioural factor most strongly related to twinning. Given the differences in age structure among different regions of the developing world, standardized figures might give a better picture of the variation among countries. However, the age standardized figures differ not much from the unstandardized ones. In only 5 of the 76 countries, the difference is more than 1 per 1000 and the largest difference is 1.4 per 1000. Hence the twinning rates presented in Figure 1 are not much affected by variation in mother's age at birth among the countries.

We also computed the number of triplets. There were 370 triplets in 2,473,209 births, or one triplet in 6,684 births. This outcome is reasonable well in line with Hellin's law [31], [32] which says that if the number of twins is one in X, the number of triplets is one in X^2^. With one in 80.05 births being a twin birth (all births in the DHS data taken together), this rule predicts the triplet incidence to be one in 6,408. This is only four percent higher than the incidence actually observed. The pattern of variation in triplet rates across the developing world is rather similar to that observed for twins. The triplet rate is 285 per million births in the high twinning countries of Africa, 155 per million births in the other African countries, 68 per million births in South and South East Asia and 83 per million births in Latin America without Caribbean. The number of quadruplets in our data is 12, which is too low to draw any meaningful conclusion.

### Trends

How did twinning rates in the major regions of the developing world vary over the last decades? The last column of Table S1 provides the answer to this question. It presents the average annual change in twinning rate between the first and the last survey for the 44 countries in our database for which data for at least two points in time are available. In Figures S1, S2, S3, and S4 the trends in these countries are displayed graphically.

The changes in twinning rates over time are generally small and not into one specific direction. On all three continents, some countries show an increase, some show a decrease and some are stable. The lack of an increase comparable to that experienced in high income countries over the last decades suggests that the influence of fertility treatments is still low in these countries. Even in India, where wealthier couples are increasingly using these techniques [33], the increase in twinning incidence over a period of 14 years was no more than 0.84 per 1000 births (14*0.06).

As the data for each point in time are based on a different DHS survey, the rather stable outcomes suggest that the twinning rates presented in this paper are measured in a reliable way. At the same time, it should be kept in mind that these are national figures and that within countries substantial variation in twinning rates among regions and groups may exist. Such sub-national variation has for example been observed between urban and rural areas in Sweden, Finland and China [7], [27], between different departments of France [1], between blacks and whites in the USA [1], [12] and between ethnic groups in Nigeria and Singapore [34], [35]. Hence changes over time in national twinning rates need not always reflect actual changes in twinning frequencies, but may to a certain extent reflect changes in fertility levels of groups or regions within countries.

## Discussion

This paper contributes to the twinning literature in several ways. First, for the first time representative and comparable national figures have become available for 75 low and middle income countries, for which earlier only non-representative figures or no figures at all were available. Second, for Asia our data reveal that twinning rates are not only low in a few countries, but in the whole Eastern, South-Eastern and Southern region. The very low twinning rate in India contradicts the idea resonating in the literature since the early 1970s [1], [5] that twinning rates among the predominantly Caucasian Indian population are at an intermediate level, comparable to that in most European countries. Third, for Africa the myth is broken that twinning rates in Nigeria are the highest in the world. High national twinning rates are found throughout Central-Africa, and in several countries twinning incidence is higher than in Nigeria. The highest national twinning rate is found is Benin (27 per 1000). Fourth, twinning rates in Latin America turn out to be at a similar low level as in Asia. Fifth, our study shows that the Demographic and Health Surveys constitute an important new data source for twin research. The DHS program is still actively running, with currently about 20 surveys in the field. Hence, in the coming years many new comparable datasets with twin data for developing countries will become available.

For twin researchers, these data are important, because in these countries natural twinning rates can still be observed. In high-income countries, the twinning picture has been altered strongly by the influence of ART. Data for African countries offer many possibilities for twin research, because of the high proportion of twins available in national representative datasets. For researchers studying monozygotic twinning, data from the low-twinning countries in Asia and Latin America may however be more interesting. With about 4 in 1000 births known to be monozygotic across the globe [1], [10], and all monozygotic twin pairs being same-sex pairs, in regions with 8 twins per 1000 births about two-third of all same-sex pairs can be expected to be monozygotic.

Regarding the quality of the data, we can conclude from the low within-country variation in twin rates that twinning information derived from DHS data is rather reliable. This does however not imply that our twinning figures are unbiased in all respects. It is important to note that these twin rates refer to live births. Stillbirths are excluded. Among stillbirths, the proportion of twins is probably somewhat higher than among live births, as fetal (and neonatal) mortality is higher among twins. It is thus possible that twin births are underreported in our data. If so, actual twinning rates will be higher than the figures presented here. Given that infant mortality rates are highest in sub-Saharan Africa, the data for Africa might be deflated most. Hence, to the extent that excluding stillbirths plays a role, the differences between twinning rates in African countries on the one hand and Asian and Latin American countries on the other might be even larger than observed in this study.

From a health policy perspective, our finding of high twinning rates throughout Central Africa point to the existence of a great public health challenge. Twins are much more vulnerable than singletons. They have lower birth weight, are more often premature, suffer more from obstetric complications, and have a much higher risk of fetal and neonatal mortality [36]–[40]. With over one in 25 children being a twin and a four times higher mortality rate among twins [37] (between a quarter and half of them does not reach age 5), the already very serious child mortality problem in the Central African region is substantially aggravated by the high number of twin births. Solving this problem calls for investments in screening programs for detecting multiple pregnancies and in facilities for antenatal, delivery, and postnatal care tailored toward the special needs of these children and their mothers [37]–[39].

## Supporting Information

Table S1 **Natural and standardized twinning rates, number of twin births, total births, survey years, and annual change for 76 low and middle income countries.**
(PDF)

Figure S1 **Twinning rates in Asian countries over time.**
(PDF)

Figure S2 **Twinning rates in Latin American countries over time.**
(PDF)

Figure S3 **Twinning rates in North and West African countries over time.**
(PDF)

Figure S4 **Twinning rates in Central and South African countries over time.**
(PDF)

## References

[1] Bulmer MG (1970). The Biology of Twinning in Man.

[2] Segal N (1999). Entwined Lives: Twins and What They Tell Us About Human Behavior.

[3] Little J, Thompson B, McGillivray I, Campbell DM, Thompson BJ (1988). Descriptive epidemiology.. Twinning and Twins.

[4] Blickstein I, Keith LG (2005). Multiple pregnancy, epidemiology, gestation & perinatal outcome, 2 ed.

[5] Nylander PPS, McGillivray I, Nylander PPS, Corney G (1975). Frequency of multiple births.. Human Multiple Reproduction.

[6] Nylander PPS, Barron SL, Thompson AM (1983). The phenomenon of twinning.. Obstetrical Epidemiology.

[7] Gan JP, Wu ZH, Tu ZM, Zheng J (2007). The comparison of twinning rates between urban and rural areas in China.. Twin Res Hum Genet.

[8] Astolfi P, Ullizi L, Zonta LA (2003). Changes in twinning rate: Italy 1950–1996.. Hum Reprod.

[9] Westergaard T, Wohlfahrt J, Aaby P, Melbye M (1997). Population based study of multiple pregnancies in Denmark, 1980–94.. BMJ.

[10] Hall JG (2003). Twinning.. Lancet.

[11] Hoekstra C, Zhao ZZ, Lambalk CB, Willemsen G, Martin NG (2008). Dizygotic twinning.. Human Reprod Update.

[12] Bortolus R, Parazzini F, Chatenoud L, Benzi G, Bianchi MM (1999). The epidemiology of multiple births.. Human Reprod Update.

[13] McGillivray I, Samphier M, Little J, Ian M, Dorris MC, McGillivray I, Campbell DM, Thompson BJ (1988). Factors affecting twinning. .. Twinning and Twins.

[14] Nylander PPS, McGillivray I, Nylander PPS, Corney G (1975). Factors which influence twinning rates.. Human Multiple Reproduction.

[15] Basso O, Nohr EA, Christensen K, Olsen J (2004). Risk of twinning as a function of maternal height and body mass index.. JAMA.

[16] Kallen K (1998). Maternal smoking and twinning.. Twin Res.

[17] Morales-Suarez-Varella MM, Bech BH, Christensen K, Olsen J (2007). Coffee and smoking as risk factors for twin pregnancies.. Twin Res Hum Genet.

[18] Murphy MF, Campbell MJ, Bone M (1989). Is there an increased risk of twinning after discontinuation of the oral contraceptive pill?. J Epidemiol Community Health.

[19] Oleszczuk JJ, Cervantes A, Kiely JL, Keith DM, Keith LG (2001). Maternal race/ethnicity and twinning rates in the United States, 1989–1991.. J Reprod Med.

[20] Pollard R (1995). Ethnic comparisons of twinning rates in California.. Hum Biol.

[21] White C, Wyshak G (1964). Inheritance in human dizygotic twinning.. New Engl J Med.

[22] Lichtenstein P, Olausson PO, Kallen AJ (1996). Twin births to mothers who are twins: A registry based study.. BMJ.

[23] Fauser BC, Devroey P, Macklon NS (2005). Multiple birth resulting from ovarium stimulation for subfertility treatment.. Lancet.

[24] Imaizumi Y (2003). A comparative study of zygotic twinning and triplet rates in eight countries, 1972–1999.. J Biosoc Sci.

[25] Blondel B, Kaminski M (2002). Trends in occurrence, determinants, and consequences of multiple births.. Semin Perinatol.

[26] Pison G, D'Addato AV (2006). Frequency of twin births in developed countries.. Twin Res Hum Genet.

[27] Eriksson AW, Fellman J (2004). Demographic analysis of the variation in the rates of multiple maternities in Sweden since 1751.. Hum Biol.

[28] Imaizumi Y, Blickstein I, Keith LG, Keith DM (2005). Demographic trends in Japan and Asia.. Multiple Pregnancy, epidemiology, gestation & perinatal outcome, 2 ed.

[29] Choi SH, Park YS, Shim KS, Choi YS, Chang JY (2010). Recent trends in the incidence of multiple births and its consequences on perinatal problems in Korea.. J Kor Med Sci.

[30] Chen CJ, Lee TK, Wang CJ, Yu MW (1992). Secular trend and associated factors of twinning in Taiwan.. Acta Genet Med Gemellol Roma.

[31] Hellin D (1895). Die Ursache der Multiparität der uniparen Tiere überhaupt und der Zwillingsschwangerschaft beim Menschen insbesondere (The causes of multiple maternities among uniparous animals and in man).. Seitz & Schauer.

[32] Fellman J, Eriksson AW (2009). On the History of Hellin's Law.. Twin Res Hum Genet.

[33] Muheree M, Sarojini NB (2006). Assisted reproductive technologies in India.. Development,.

[34] Nylander PPS (1971). Ethnic differences in twinning rates in Nigeria.. J Biosoc Sci.

[35] Chia KS, Lee JJM, Cheung P, Cheung KH, Seielstad M (2004). Twin births in Singapore: A population-based study using the national birth registry.. An Acad Med Singapore.

[36] Wedstrom KD, Gall SA (1988). Incidence, morbidity and mortality, and diagnosis of twin gestations.. Clin Perinatol.

[37] Pison G, Pison G, Van de Walle E, Sala-Diakanda M (1989). Les jumeaux: Frequence, status social et mortalite.. Mortalite et societe en Afrique au sud du Sahara.

[38] Katz J, West KP, Kathry SK, LeClerq S, Christian P (2001). Twinning rates and survival of twins in rural Nepal.. Int J Epidemiol.

[39] Igberase GO, Ebeigbe PN, Bock-Oruma A (2008). Twinning rate in a rural mission tertiary hospital in the Niger delta, Nigeria.. Journal of Obstet and Gynaecol.

[40] Rutstein SO (1983). Infant and child mortality levels, trends and demographic differentials: WFS Comparative Studies No. 24.

