# Supplementary material for: Twinning across the Developing World
Source: PLoS One. 2011 Sep 28;6(9):e25239. doi: 10.1371/journal.pone.0025239 (PMC3182188; doi:10.1371/journal.pone.0025239)
Supplement: Table S1 — Natural and standardized twinning rates, number of twin births, total births, survey years, and annual change for 76 low and middle income countries. (PDF) [file pone.0025239.s001.pdf]

Table S1. Natural and standardized twinning rates, number of twin births, total births, survey years, and annual change for 76 low and middle income countries

Country	Continent	Natural Twinning rate[a]	Standardized Twinning rate[b]	Twin Births[c]	Total Births[d]	First Year[e]	Last Year[f]	Annual Change[g]
Albania	Europe	12.0	11.6	49	4079	2009	-	-
Armenia	Asia	9.0	10.1	69	7641	2000	2005	0.11
Azerbaijan	Asia	7.1	7.9	36	5046	2006	-	-
Bangladesh	Asia	8.5	9.8	359	42142	1996	2007	-0.03
Benin	Africa	27.9	27.7	1495	53595	1996	2006	-0.23
Bolivia	Lat. America	6.7	6.5	383	57081	1998	2008	-0.01
Brazil	Lat. America	8.8	9.3	98	11099	1996	-	-
Burkina Faso	Africa	16.1	15.9	750	46453	1993	2003	0.25
Cambodia	Asia	9.4	8.6	379	40239	2000	2005	-0.20
Cameroon	Africa	21.9	22.9	515	23522	1998	2004	0.50
Central African Rep.	Africa	13.8	14.3	116	8418	1996	-	-
Chad	Africa	15.6	16.1	409	26246	1997	2004	-0.23
Colombia	Lat. America	8.2	8.4	409	49876	1995	2005	-0.04
Comoros	Africa	19.9	18.9	83	4164	1996	-	-
Congo Brazzaville	Africa	21.3	21.9	195	9134	2005	-	-
Congo Dem. Rep.	Africa	18.2	17.9	308	16933	2007	-	-
Cote d'Ivoire	Africa	15.9	16.3	292	18384	1994	1999	0.35
Dominican Rep.	Lat. America	11.2	12.0	650	57906	1996	2007	-0.07
Ecuador	Lat. America	9.6	9.8	59	6167	1987	-	-
Egypt	Africa	17.7	17.5	2007	113323	1995	2008	0.16
Ethiopia	Africa	11.0	10.9	474	43092	2000	2005	-0.33
Gabon	Africa	19.4	20.0	173	8936	2000	-	-
Ghana	Africa	20.4	19.0	431	21078	1998	2008	0.40
Guatemala	Lat. America	7.3	7.2	217	29911	1995	1999	-0.48
Guinea	Africa	21.3	21.1	572	26846	1999	2005	1.05
Haiti	Lat. America	14.1	13.3	477	33861	1994	2005	-0.31
Honduras	Lat. America	8.1	8.5	206	25530	2005	-	-
India	Asia	7.2	7.7	2685	373093	1992	2006	0.06
Indonesia	Asia	7.2	7.1	799	110601	1997	2007	0.08
Jordan	Asia	14.4	13.1	491	34203	2002	2007	0.09
Kazakhstan	Asia	9.8	9.7	66	6740	1995	1999	0.39
Kenya	Africa	15.6	16.1	747	47804	1993	2008	-0.04
Kyrgyzstan	Asia	8.7	8.9	38	4348	1997	-	-
Lesotho	Africa	14.5	14.6	209	14414	2004	2010	-0.29
Liberia	Africa	20.1	20.6	222	11026	2007	-	-
Madagascar	Africa	10.6	10.7	620	58512	1992	2009	-0.05
Malawi	Africa	20.8	21.6	1102	53030	1992	2004	-0.36
Maldives	Asia	8.0	8.2	59	7352	2009	-	-
Mali	Africa	16.9	17.3	1309	77406	1995	2006	0.12
Mauritania	Africa	17.2	16.7	162	9435	2001	-	-
Mexico	Lat. America	10.5	10.7	112	10700	1987	-	-
Moldova	Europe	8.2	8.9	27	3294	2005	-	-
Morocco	Africa	12.1	11.1	297	24598	1992	2003	0.29
Mozambique	Africa	18.6	19.2	645	34702	1997	2003	0.23
Namibia	Africa	13.3	13.0	253	18977	2000	2006	0.08

Table continued on next page

Table S1 continued

Country	Continent	Natural Twinning rate[a]	Standardized Twinning rate[b]	Twin Births[c]	Total Births[d]	First Year[e]	Last Year[f]	Annual Change[g]
Nepal	Asia	6.9	7.2	287	41471	1996	2006	0.07
Nicaragua	Lat. America	8.1	8.4	268	33228	1998	2001	0.27
Niger	Africa	17.3	17.8	595	34433	1998	2006	0.06
Nigeria	Africa	19.0	19.0	1581	83003	1999	2008	-0.30
Pakistan	Asia	9.6	9.5	323	33483	1991	2007	0.10
Paraguay	Lat. America	9.9	9.7	85	8611	1990	-	-
Peru	Lat. America	7.6	7.4	804	105340	1996	2008	0.01
Philippines	Asia	7.2	6.8	329	45516	1998	2008	0.03
Rwanda	Africa	12.3	11.1	542	44135	1992	2005	0.32
Sao Tome & Principe	Africa	19.3	18.9	69	3579	2009	-	-
Senegal	Africa	15.1	15.0	500	33166	1992	2005	0.35
Sierra Leone	Africa	17.7	17.9	213	12030	2008	-	-
South Africa	Africa	12.6	12.5	133	10552	1998	-	-
Sri Lanka	Asia	9.4	9.0	79	8422	1987	-	-
Sudan	Africa	14.9	14.8	196	13187	1990	-	-
Swaziland	Africa	14.3	14.9	81	5673	2006	-	-
Tanzania	Africa	18.8	18.9	717	38232	1992	2004	0.32
Thailand	Asia	6.8	6.8	53	7825	1987	-	-
Timor Leste	Asia	8.9	8.5	188	21119	2009	-	-
Togo	Africa	21.4	21.3	301	14074	1998	-	-
Trinidad & Tobago	Lat. America	7.2	7.4	28	3883	1987	-	-
Tunisia	Africa	14.3	13.3	131	9146	1988	-	-
Turkey	Asia	9.9	9.8	264	26600	1993	2003	0.01
Uganda	Africa	14.6	15.4	630	43078	1995	2006	0.10
Ukraine	Europe	8.9	8.2	24	2711	2007	-	-
Uzbekistan	Asia	9.8	9.9	49	4985	1996	-	-
Vietnam	Asia	6.2	6.5	83	13354	1997	2002	-0.39
Yemen	Asia	9.8	9.0	169	17223	1991	-	-
Zambia	Africa	18.6	19.2	718	38675	1996	2007	-0.11
Zimbabwe	Africa	15.7	16.0	401	25538	1994	2006	-0.19
Total without China	75 countries	13.2[h]	13.2 [h]	30,895	2,473,209	1987	2010	-
China	Asia	7.9	8.7	186,273	23,477,961	1989	-	-
Total with China	76 countries	13.1[i]	13.1[i]	217,168	25,951,170	1987	2010	-

Notes
a Natural twinning rate=1000*twin births/total births.
b Standardized for age at birth of the mother.
c Number of twin births in ten years before survey.
d Total number of live births in ten years before survey .
e Year of first survey.
f Year of last survey (only available for countries with at least two surveys).
g Average annual change in twinning rate between first and last survey.
h Average twinning rate 75 countries, each country weighted equally
i Average twinning rate 76 countries, each country weighted equally
