# Supplementary material for: Twinning across the Developing World
Source: PLoS One. 2011 Sep 28;6(9):e25239. doi: 10.1371/journal.pone.0025239 (PMC3182188; doi:10.1371/journal.pone.0025239)
Supplement: Figure S3 — Twinning rates in North and West African countries over time. (PDF) [file pone.0025239.s004.pdf]

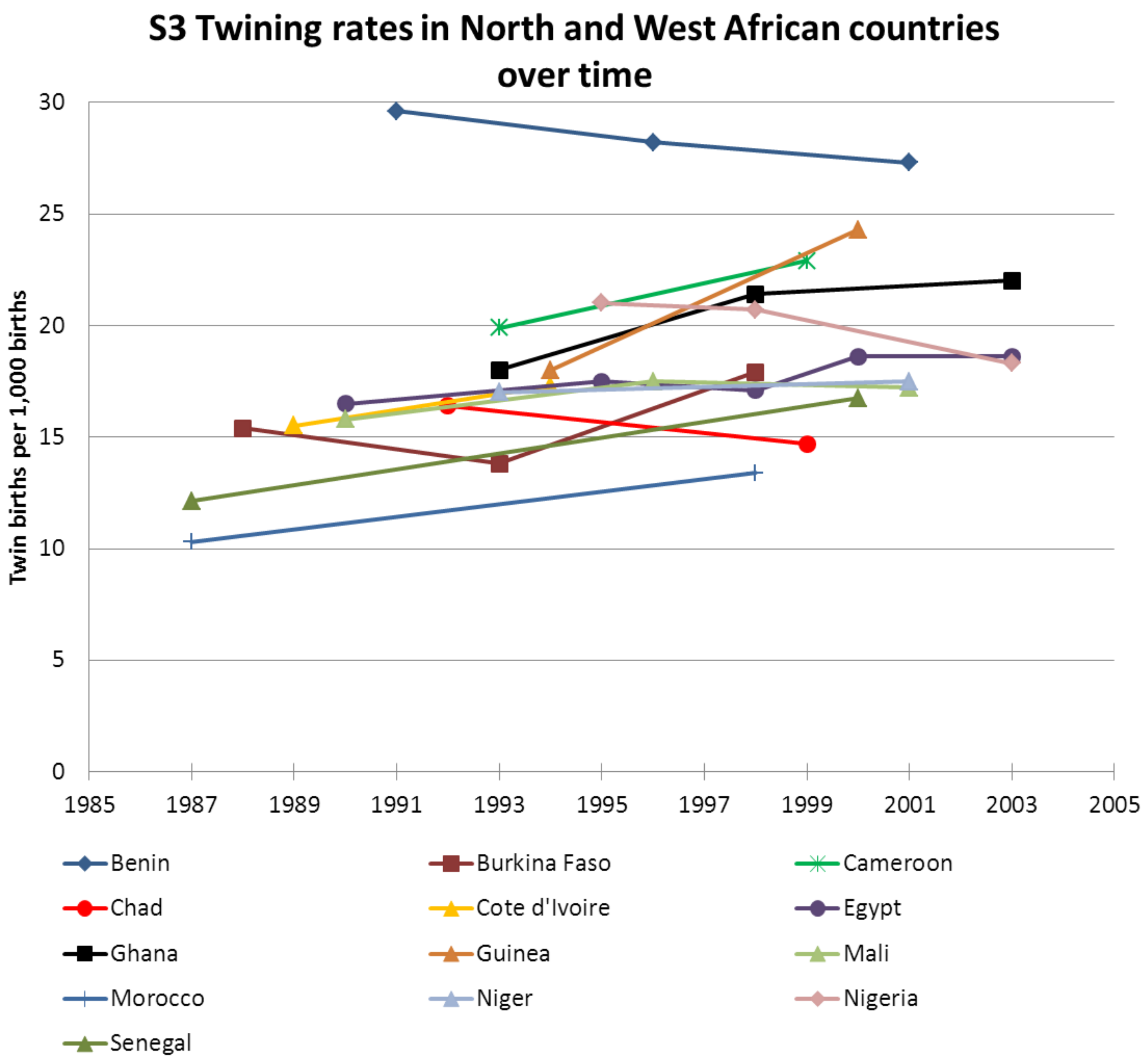


Note. Years on the x-axis refer to the mid-point of the ten year period in which births are observed (for example, 1996 refers to the 1991-2001 period).
